# Game Performance Versus Competitive Performance in the World Championship of Handball 2011

**DOI:** 10.2478/hukin-2013-0014

**Published:** 2013-03-28

**Authors:** Óscar Gutiérrez, José L. Ruiz

**Affiliations:** 1 Center of Research in Sport, University Miguel Hernández of Elche, Spain.; 2 Center of Operations Research, University Miguel Hernández of Elche, Spain.

**Keywords:** team game performance, Data Envelopment Analysis, cross-efficiency evaluation, handball

## Abstract

This article assesses the game performance of the teams participating in the Men’s World Championship of Handball of 2011 by using Data Envelopment Analysis (DEA) and the cross-efficiency evaluation. DEA uses Linear Programming to yield a measure of the overall performance of the game of particular teams, and allows us to identify relative strengths and weaknesses by means of benchmarking analysis. The cross-efficiency evaluation provides a peer-appraisal of the teams with different patterns of game, and makes it possible to rank them. Comparisons between this ranking and the final classification in the championship provide an insight into the game performance of the teams versus their competitive performance. We highlight the fact that France, which is the world champion, is also identified as an “all-round” performer in our game performance assessment.

## Introduction

Tactics are considered an important aspect of team sports, which can be expressed individually or collectively. The collective strategic behaviour is often understood as the sum of individual behaviours, thus tactical decisions are sometimes evaluated individually as a way of evaluating collective tactics. The collective game in team sports is developed by taking into account the characteristics of the team’s own players and the need to counteract the quality of the players of the opposing team. The achievement of high team performance depends on several factors such as technical skills, physical fitness or relationships between players. This is a complex system that is constantly changing and cannot be controlled by means of external fixed criteria, so regulatory mechanisms of dynamical systems can facilitate this task.

Tactical assessment can be made on the basis of either real matches or scrimmage games, with the purpose of evaluating specific aspects of tactical decisions of the players. Nevertheless, the assessment of tactics in real matches is very important, because there are situations that only arise in the context of real game and these would be difficult to reproduce in scrimmage conditions. Extensive literature deals with tactical performance evaluation in team sports based on tactical indexes. There are several articles that are intended to determine which indexes are more representative or which may be more significant for the analysis of tactics. See the investigations in football (Tenga et al., 2010a, 2010b, 2010c), basketball (Swalgin, 1998; Trninic and Dizdan, 2000; Trninic et al., 2000) and water polo (Hraste et al., 2008).

There are also some works focusing on different strategies, approaches and styles of the teams, which may lead to different tactical indexes. It is argued that the analysis of the different groups formed in the preliminary stages of the Handball World Championship 2003 generated different indicators of success taking into account the reference groups and the different characteristics of each team (Gruic et al., 2006). This suggests that we should not use a unique pre-established pattern of game that is imposed to all the teams in their assessments. Instead, we should use a model that somehow takes into consideration the characteristics of the game of each team. This is why we propose the use of “Data Envelopment Analysis” (DEA) (Charnes et al., 1978) for the assessment of team game performance.

To define a measure of the overall team performance in the game we must determine how the variables that describe the different aspects of the game have to be aggregated. In order to do so, we need to specify the importance (the weight) that is to be attached to each of these aspects of the game. Traditionally, teams are assessed on the basis of a common set of weights. However, in this traditional approach the choice itself of the weights often raises serious difficulties, and in many cases the analysts do not agree upon the weights to be used. In DEA there is no need to know such weights beforehand. The weights are determined trying to show the team under assessment in its best possible light. Besides, the DEA weights are team-specific, so this methodology provides a self-evaluation in which each team can exploit its strengths in the assessments. As another interesting feature of this methodology, we point out that with DEA we may develop plans for improvement of the game by means of benchmarking analysis. The teams are classified into efficient and inefficient, so the latter are assessed with respect to the former. DEA allows us to identify the weaknesses in the game of the inefficient teams and to set efficient targets, which represent levels of performance in each aspect of the game that would make each of them perform efficiently. Like the DEA weights, the targets are also team-specific. These targets result from the selection of a benchmark that is made taking into consideration the type of the game of the team under assessment. The key issue is that each team may have a different way to achieve the efficiency, which will obviously depend on the characteristics of its game. However, the main weakness of DEA is perhaps the fact that it cannot provide a ranking of teams based on the measures of efficiency it yields since, as said before, the score of each team is calculated with weights that are usually different from those of the others. For this reason, we also propose here the use of the cross-efficiency evaluation (Sexton et al., 1986; Doyle and Green, 1994), which is an extension of DEA aimed at providing a ranking. The idea behind the cross-efficiency evaluation is to assess each team with the DEA weights of all the teams instead of with only its own weights. This provides a peer-appraisal of the game performance of the teams with different patterns of game and, in addition, we can rank the teams according to the resulting cross-efficiency scores.

DEA has been successfully used in public and private sectors and, in particular, in the context of sports. For instance, Cooper et al. (2009) assess basketball players by using the statistics of the Spanish premier league. Cooper et al. (2011) provide a ranking of basketball players with a cross-efficiency evaluation. DEA and cross-efficiency evaluation are combined for the assessment and ranking of professional tennis players (Ruiz et al., 2011). Ramón et al. (2012) also rank tennis players with a common set of weights obtained from DEA weights. See also the evaluations of players by using DEA in baseball (Anderson and Sharp, 1997; Chen and Johnson, 2010; Sexton and Lewis, 2003; Sueyoshi et al., 1999), golf (Fried et al., 2004; Fried and Tauer, 2011; Ueda and Amatatsu, 2009) and football (Alp, 2006).

DEA has been used not only for the assessment of players. See, for example, the case of soccer, where we can find evaluations of teams (Boscá et al., 2009; Espitia-Escuer and García-Cebrián, 2004; García-Sánchez, 2007; González-Gómez and Picazo-Tadeo, 2010; Haas, 2003; Haas et al., 2001), coaches (Dawson et al., 2000) and clubs (Barros et al., 2010). At the level of countries, DEA has been used for measuring the performance of the participating nations at the Summer Olympics Games (Lozano et al., 2002; Soares de Mello et al., 2009; Wu et al., 2010; Zhang et al., 2009).

Finally, we can also find applications of DEA analyzing the efficiency in sports from other perspectives. Fizel and D’Itri (1999) study the impact on organizational performance of practices like firing and hiring managers. Volz (2009) provides efficiency scores not only of team performance, but also of player salaries in Major League Baseball, and Einolf (2004) measures franchise payroll efficiency in the National Football League and Major League Baseball.

As far as we know, DEA and cross-efficiency evaluation have not yet been used in handball. In this paper, we illustrate their use in an assessment of team game performance of the nations participating in the Men’s World Championship in 2011 based on the statistics reported in that tournament. The comparisons of the results obtained with the final classification in the championship provide an insight into the game performance of the teams versus their competitive performance.

## Material and Methods

### Participants

The 24 teams that played in the Men's World Handball Championships of 2011 in Sweden were included.

### Measures

The data in this article have been taken directly from the official statistics of the International Handball Federation (IHF) without elaboration by the authors. These are available in http://www.ihf.info/, and include all of the matches played during the Men's World Handball Championships of 2011 held in Sweden. Thus, we have a sample of 24 national teams which are described in terms of the following 8 variables: G6m (y_1_), Gwing (y_2_), G9m (y_3_), G7m (y_4_), Gfastb (y_5_) and Gbt (y_6_) which are, respectively, the number of goals per game scored from 6m, from the wing position, those scored from 9m and 7m and the number of fastbreak and breakthrough goals, in all cases adjusted by the percentage of success; Rec (y_7_) is the number of recoveries per game and Bloc (y_8_) is the number of blocks per game. These data provide information of each team regarding goals and shots from different distances, situations and positions, recoveries and blocks, and may thus reflect the effects of the tactical decisions concerning different aspects of the game like shooting, both in a positional attack and in transition, and defense. The 24 teams can be therefore described by means of the output vectors

$${\text{P}}_{\text{j}}={\left({\text{y}}_{1,\text{j}},\ldots ,{\text{y}}_{8,\text{j}}\right)}^{\prime },\text{j}=1,\ldots ,24.$$

### Analysis

We use the so-called CCR DEA model for the analysis of team efficiency. For a given team, say team 0, the following linear problem provides the weights that allows us to aggregate the information regarding the 8 outputs above into a single value θ_0_

(1) $$\begin{array}{ll} \text{Maximize} & \theta_{0}=\omega_{1}\times y_{1,0}+\ldots +\omega_{8}\times y_{8,0}\\ \text{subject}\;\text{to}: & \\ & \omega_{1}\times y_{1,1}+\ldots +\omega_{8}\times y_{8,1}\leq 1\\ & \ldots \ldots \ldots \ldots \ldots \ldots \ldots \ldots \ldots \ldots \\ & \omega_{1}\times y_{1,24}+\ldots +\omega_{8}\times y_{8,24}\leq 1\\ & \omega_{8},\ldots ,\omega_{8}\geq 0 \end{array}$$

We can see that the objective in (1) is to find the weights ω’s that maximize the corresponding weighted sum of outputs for team 0, subject to the condition that this weighted sum, calculated with these weights for the rest of teams, is in all cases lower than or equal to a given value, which is usually set at 1. Thus, team 0 is said to be efficient if θ_0_=1. Otherwise, it is inefficient, and the lower θ_0_ the lesser its efficiency. Looking at model (1) we realize that in DEA there is no need to a priori know the weights that represent the importance to be attached to the different aspects of the game. When solving (1) each team has total freedom in the choice of such weights, which are determined trying to show it in its best possible light. This is of particular interest in the identification of inefficient teams: if a team is free to choose its own weights and others have a higher efficiency score with those weights, then a stronger statement is being made. However, this total weight flexibility may become an issue in the identification of efficient teams, since they sometimes take advantage of it and achieve the efficiency with weights that are inconsistent with the accepted views of experts. In particular, in DEA the units under assessment sometimes achieve the efficiency ignoring the variables with poor performance by attaching them a zero weight. To avoid this, it has been proposed in the literature to restrict the weights by incorporating into the analysis value judgements from experts regarding the relative importance of the variables (see chapter 4 in Cooper et al., 2011, for a recent survey on choices and uses of DEA weights). To be specific, in the analysis in the present paper we have imposed that the importance attached to the variables concerned with defense cannot be larger than that of those regarding the offensive aspects of the game.

By virtue of the duality theory in linear programming, DEA also provides a benchmarking analysis by solving the following model

(2) $$\begin{array}{ll} \text{Maximize} & \phi_{0}\\ \text{subject}\;\text{to}: & \\ & \lambda_{1}\times {\text{P}}_{1}+\ldots +\lambda_{24}\times {\text{P}}_{24}\geq \phi_{0}\times {\text{P}}_{0}\\ & \lambda_{1}+\ldots +\lambda_{24}=1\\ & \lambda_{1},\ldots ,\lambda_{24}\geq 0 \end{array}$$

**Figure 1:**
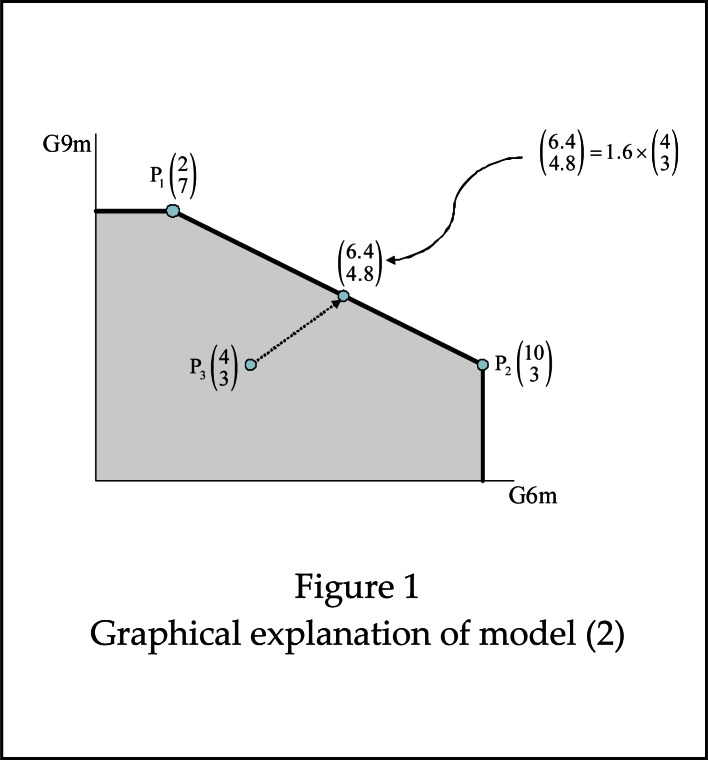
Graphical explanation of model (2)

The optimal value of (2), ϕ_0_, is actually the inverse of θ_0_ in (1). Therefore, team 0 is efficient if ϕ_0_ = 1, while it is rated as inefficient if ϕ_0_ > 1. Figure 1 illustrates graphically the idea behind model (2). Suppose that we have 3 handball teams that are to be assessed regarding two game factors, say, G6m and G9m. Their records in the championship in these two variables are P_1_(2,7) for team 1, P_2_(10,3) for team 2 and P_3_(4,3) for team 3, i.e., team 1, for example, scored 2 6m goals per game and 7 9m goals per game, and so on. The grey area is the so-called production possibility set (PPS), and includes the teams (real or virtual) that are assumed to be potential benchmarks in the assessments. Roughly speaking, in the PPS we have combinations of real teams, and others that represent worse performances. The points on the frontier of the PPS (the bold line) represent obviously “best practice” performances. Teams 1 and 2 are rated as efficient because we cannot find in the PPS other teams that score more 6m goals and more 9m goals than them. In that case, ϕ_1_ and ϕ_2_ cannot be greater than 1. However, team 3 is inefficient because other teams in the PPS outperform it regarding these two game factors. In particular, the point (6.4,4.8) shows that team 3 should score 6.4 6m goals and 4.8 9m goals in order to perform at the levels of the efficient teams (these are actually the targets for team 3). In other words, ϕ_3_ = 1.60 is the efficiency score of team 3, which means that it should improve by 60% in these two game factors. The point (6.4,4.8) is a benchmark for team 3 that results from a combination of team 1 and team 2 in which the participation of the former is 45% and that of latter is 55%, i.e., λ_1_=0.45 and λ_2_=0.55 in model (2) (obviously, λ_3_=0), so that

$$\left(\begin{array}{l} 6.4\\ 4.8 \end{array}\right)=0.45\times {\text{P}}_{1}+0.55\times {\text{P}}_{2}.$$

Finally, we use the cross-efficiency evaluation for the ranking of teams. The cross-efficiencies of team 0 are the assessments of this team with the weights of the others. That is, if $\left(\omega_{1}^{\text{d}},\ldots ,\omega_{8}^{\text{d}}\right)$ are the weights of team d, obtained by solving (1) for that team, then the cross-efficiency

(3) $${\text{E}}_{\text{d},0}=\frac{1}{\omega_{1}^{\text{d}}\times {\text{y}}_{1,0}+\ldots +\omega_{8}^{\text{d}}\times {\text{y}}_{8,0}}$$

is an evaluation of team 0 with the weights of team d. The cross-efficiency score of team 0 is the average of such cross-efficiencies, i.e.,

(4) $${\bar{\text{E}}}_{0}=\frac{1}{24}\left({\text{E}}_{1,0}+\ldots +{\text{E}}_{24,0}\right)$$

The cross-efficiency scores provide thus a peer-appraisal in which each team is assessed with reference to the different patterns of game that the different teams have used in their DEA assessments, and also determine a full ranking of teams.

For those readers interested in details on the DEA models, their formulations and properties, see the textbook by Cooper et al. (2007).

## Results

The DEA model revealed that 9 out of the 24 teams participating in the championship were efficient. For each of them, Table 1 records the contributions to the efficiency of each game factor. These contributions, which are called “virtual weights”, are the product of the absolute weights and the corresponding actual values, i.e., for team 0 these would be ω_r_ × y_r0_, r=1,..,8, where the ω’s are the weights provided by (1) when solved for that team. They are dimensionless and represent the percentages of contribution of each factor to the total efficiency (100%), so they can be seen as the relative importance attached to each aspect of the game in the assessment of each team. This table also reports the number of times each of the efficient teams acted as referent in the assessment of the inefficient ones, which is determined as the number of times the corresponding λ_j_ in model (2) is non-zero in the assessment of the different teams.

The benchmarking analysis provided by DEA is reported in Table 2. For each inefficient team, in this table we have its actual data (in the first row of each team) and the corresponding efficient targets (in the second row). The third row records the difference between the target and the actual data in relation to the actual data. Large values of these percentages may suggest the need of the team under assessment for improvement in the corresponding aspect of the game. Table 2 also reports which efficient teams compose the benchmark used in the assessments, together with their contributions as efficient referents in such benchmark, i.e., the λ_j_’s provided by model (2).

Table 3 records the cross-efficiencies (3) and the cross-efficiency scores (4). We note that in our analysis we used a variant of the standard cross-efficiency evaluation that assesses the teams by only using the weights of those that have been rated as efficient in the DEA self-evaluation (Ramón et al., 2011). Thus, the rows of this table correspond to each of the teams participating in the championship, and in each of them we have the evaluations of their game (the cross-efficiencies) with the weights of each of the efficient teams (under the corresponding column). The last column of the table shows the cross-efficiency scores and in brackets their corresponding rankings. We can see, for instance, that France ranks 1^st^ followed by Spain, Denmark and Slovakia, in this order. The teams in the rows of the table appear in order of the final classification of the world championship, so we can make comparisons between the two rankings.

## Discussion

On many occasions, tactics are validated on the basis of the achievement of victory, the winning team being rated as the best. However, we should not close the door to the analysis of other teams whose performance can serve as a model of efficiency for the game. For example, Table 1 shows that the 9 efficient teams achieved the efficiency with different patterns of game. We can see that France used a pattern of game in which all of the factors considered have the same importance. This shows a good performance of France in all of the aspects of the game. Denmark and Spain needed to put more weight in some of the game factors in order to be rated as efficient. Table 1 reveals that Denmark exploited to some extent its relative strength in G6m, Gwing and G9m in the achievement of the efficiency (with a contribution to the efficiency of 22.79%), while Spain did the same with Gwing (14.40%), G7m and Rec (both with a contribution of 26.99%). We can also see in this table that these three teams played an important role as benchmarks for the remaining players: they acted as referents in the assessments of 13, 9 and 13 inefficient teams, respectively.

In contrast, other teams like Iceland, Hungary, Norway and Korea achieved the efficiency with a very specialized pattern of game. See, in particular, the case of Norway, whose efficiency was due mainly to exploiting its good performance in G6m (with a contribution to the efficiency of 68.03%), or that of Korea, which exploited to a large extent its behavior in G7m (54.79%), and also in Gbt (32.65%). We also note that neither Korea nor Hungary were referents for any of the inefficient teams.

Concerning the inefficient teams, Table 2 provides useful information for benchmarking purposes. For example, we can see that Germany is very similar to its benchmark, which is a virtual team determined mostly by France (43.24%) and Denmark (24.19%) (also by Spain and Norway with a less relevant role). Its actual data are very close to the targets provided in most of the aspects of the game. However, the DEA model identifies three areas for potential improvement: G7m, where the actual data is 1.46 whereas the corresponding target is 2.14, which gives rise to a potential of improvement of 46.71%; Gfastb, where it is needed a raise from the actual 2.86 to the target 4.32 which means an improvement of 50.75%; and also in Bloc, where the percentage of improvement is 28.77%. Poland, whose benchmark is mainly determined by France (48.94%) and Norway (43.14%), also shows a good performance in several of the factors of the game, while at the same time there is some room for improvement in other factors like G9m, Gwing, Gbt and G7m. That could be also the case of Austria or Brazil, but for these teams some important weaknesses are detected: in Gbt for Austria, with a percentage of improvement of 101.51%, and in G9m (68.12%) and in Gfastb (53.15%) for Brazil. Finally, teams like Australia or Algeria exhibit a poor performance in practically all of the aspects of the game.

As for the cross-efficiency evaluation, we can firstly see in the row of France in Table 3 that all cross-efficiencies equal 1, which means that France is rated with the maximum efficiency with the patterns of game that all the efficient teams used in their assessments. In other words, the cross-efficiency evaluation identifies France as an “all-round” performer, because it is rated as efficient with a wide variety of models of game. As a result, it ranks 1^st^: its cross-efficiency score 1 is the largest in the last column of this table. Denmark and Spain are also rated as efficient with the weights of some other teams. France, Spain, Croatia and Slovakia assess Denmark as efficient (aside from Denmark itself), while Spain is evaluated as efficient by Hungary, Korea and Slovakia (apart from by Spain itself). However, other countries give these two teams some poor assessments: Korea and Iceland give Denmark the scores 1.34 and 1.20, respectively, perhaps due to fact that they both use a very specialized pattern of game, while Norway and Denmark make a similar evaluation of Spain. In the case of Norway, the reason behind the low score (1.38) can also be the specialization of the Norway’s game model above mentioned, but in that of Denmark it seems that this team is penalizing Spain (1.21) in forcing it to put more weight on G9m and G6m, which are weaker points of Spain’s game. Finally, we can see that all of the teams give good ratings to Slovakia, and this is why it eventually ranks 2^nd^, together with Denmark and Spain.

The cross-efficiency evaluation has made it possible to discriminate between the teams initially rated as efficient in the DEA self-evaluation. Note that the cross-efficiency score of the teams in the first five positions of the ranking (Croatia is the country ranking 5^th^) are substantially larger than those of the other four, which are the efficient teams that have used the most unbalanced patterns of game in their assessment (Norway, Iceland, Korea and Hungary). Perhaps as a result, some inefficient teams like Poland and Brazil rank before these four efficient teams.

The comparison between the ranking concerning game performance provided by the cross-efficiency evaluation and the final classification of the championship allows us to conclude that France, which is the world champion, is an “all-round” performer, now in the sense that it is the best regarding both game performance and competitive performance. Denmark and Spain, which were 2^nd^ and 3^rd^ in the tournament, respectively, keep their positions in our analysis, so they are also good performers. However, we can also see differences between both rankings. Among them, we highlight the cases of Slovakia and Brazil on one hand and that of Sweden on the other. While in the ranking provided by the cross-efficiency evaluation Slovakia and Brazil gain 15^th^ and 14^th^ positions, respectively, with respect to the final classification in the world championship, Sweden would lose 13^th^. Thus, we can conclude that Brazil and Slovakia did not exploit sufficiently in competition the good performance of their game, whereas Sweden showed itself as a strong competitor. It should be noted that Brazil and Slovakia had poor results in the first round of the championship, when they had to play against teams with more potential. In contrast, we would like to stress the fact that Sweden hosted the Championship, so the emotional factor or the home advantage may have given them some edge, and this might explain their good results in competition when those concerned with the performance of the game are not particularly good.

## Conclusions

This paper illustrates the use of DEA and cross-efficiency evaluation for the assessment of game performance of sports teams. In particular, this study could be useful for coaches of handball teams to improve individual and collective tactics in competition. There are other issues that can also be addressed with these methodologies. For example, although we leave out of consideration such issues like team budgets, etc., the DEA models can incorporate that type of inputs in their formulations, if available, and develop measures of team efficiency. This could also be considered in assessments of performance at the level of players. Likewise, these assessments can be made from a different perspective like that concerned with organizational performance. In general, the results obtained have shown that DEA and cross-efficiency evaluation are useful support tools for coaching and managing sports teams.

## Figures and Tables

**Table 1:** Efficient teams: Contributions to the efficiency and number of times acting as referent

TEAM	G6m	Gwing	G9m	G7m	Gfastb	Gbt	Rec	Bloc	total	#ref.
FRA	11.04%	11.04%	11.04%	13.38%	13.38%	13.38%	13.38%	13.38%	100.00%	13
DEN	22.79%	22.79%	22.79%	6.32%	6.32%	6.32%	6.32%	6.32%	100.00%	9
SPA	6.32%	14.40%	6.32%	26.99%	6.32%	6.32%	26.99%	6.32%	100.00%	13
CRO	5.28%	23.80%	5.28%	24.90%	5.28%	24.90%	5.28%	5.28%	100.00%	2
ICE	3.13%	3.13%	3.13%	3.13%	40.61%	3.13%	40.61%	3.13%	100.00%	5
HUN	1.95%	1.95%	1.95%	35.42%	48.64%	1.95%	1.95%	6.18%	100.00%	0
NOR	68.03%	1.80%	1.80%	1.80%	1.80%	1.80%	1.80%	21.17%	100.00%	6
KOR	6.64%	1.18%	1.18%	54.79%	1.18%	32.65%	1.18%	1.18%	100.00%	0
SVK	34.46%	8.43%	4.75%	33.39%	4.75%	4.75%	4.75%	4.75%	100.00%	4

**Table 2:** Benchmarking analysis: Actual data and efficient targets (inefficient teams)

TEAM	G6m	Gwing	G9m	G7m	Gfastb	Gbt	Rec	Bloc	Benchmarks
SWE	2.44	2.50	3.30	1.58	3.29	3.31	3.50	2.90	FRA(28.56%),SPA(24.23%),DEN(17.45%),CRO(29.77%)
	3.42	2.93	4.05	2.33	3.86	3.88	4.11	4.14	
	40.37%	17.35%	22.83%	47.87%	17.35%	17.35%	17.35%	42.90%	

POL	4.12	1.61	3.58	1.88	4.20	2.48	4.00	4.11	FRA(48.94%),SPA(6.91%),NOR(43.14%),ICE(1.01%)
	4.30	1.92	4.47	2.12	4.38	2.91	4.17	4.28	
	4.22%	18.83%	24.74%	12.66%	4.22%	17.15%	4.22%	4.22%	

SRB	2.50	3.13	4.27	2.03	2.24	2.79	2.11	3.11	FRA(23.78%),SPA(24.20%),DEN(45.72%),CRO(6.31%)
	3.57	3.44	4.69	2.23	4.09	3.07	3.99	4.11	
	42.76%	9.82%	9.82%	9.82%	82.63%	9.82%	89.11%	32.12%	

GER	3.88	2.63	4.71	1.46	2.86	2.73	4.00	3.33	FRA(43.24%),SPA(13.24%),DEN(24.19%),NOR(19.33%)
	3.94	2.67	4.78	2.14	4.32	3.12	4.06	4.29	
	1.44%	1.44%	1.44%	46.71%	50.75%	14.14%	1.44%	28.77%	

ARG	2.43	2.02	2.28	2.13	3.46	3.11	4.11	2.89	FRA(47.24%),SPA(43.70%),DEN(8.11%),ICE(0.95%)
	3.40	2.38	4.30	2.52	4.09	3.68	5.01	3.87	
	40.29%	18.24%	88.04%	18.24%	18.24%	18.24%	21.86%	34.05%	

EGY	2.60	3.18	2.30	1.72	2.48	1.68	3.29	2.71	SPA(36.05%),DEN(63.95%)
	3.54	3.93	4.53	2.31	3.93	2.26	4.06	3.79	
	35.74%	23.61%	96.99%	34.06%	58.75%	34.30%	23.61%	39.53%	

ALG	2.55	1.50	2.62	0.57	2.10	1.98	3.29	2.14	FRA(31.80%),SPA(39.55%),DEN(3.69%),NOR(21.87%),SVK(3.09%)
	3.79	2.23	3.89	2.49	4.00	2.95	4.88	3.73	
	48.53%	48.53%	48.53%	336.20%	90.39%	48.53%	48.53%	74.15%	

JPN	2.22	2.15	1.76	0.86	4.71	3.62	3.00	1.29	FRA(63.88%),DEN(13.90%),ICE(22.22%)
	3.70	2.27	5.13	1.98	4.96	3.81	4.13	4.24	
	66.69%	5.21%	191.49%	129.84%	5.21%	5.21%	37.79%	229.42%	

AUT	3.72	1.26	4.52	1.56	4.99	2.04	3.29	4.29	FRA(75.54%),NOR(2.79%),ICE(21.67%)
	3.73	1.87	5.09	2.01	4.99	4.11	4.31	4.29	
	0.14%	48.71%	12.65%	29.53%	0.14%	101.51%	31.28%	0.14%	

ROU	2.57	1.80	4.02	2.03	3.65	2.54	4.57	4.00	FRA(69.66%),SPA(30.34%)
	3.49	2.09	4.67	2.39	4.31	4.26	4.84	4.23	
	35.85%	16.31%	16.18%	17.66%	18.22%	67.84%	5.81%	5.81%	

TUN	3.11	2.11	3.11	1.08	2.16	1.66	3.00	3.00	FRA(17.98%),SPA(17.10%),DEN(29.12%),NOR(34.35%),SVK(1.45%)
	4.15	2.82	4.33	2.18	4.13	2.21	4.00	4.00	
	33.45%	33.45%	39.30%	102.06%	91.19%	33.45%	33.45%	33.45%	

BRA	4.13	3.36	2.06	2.24	2.67	2.48	3.57	3.14	SPA(16.38%),DEN(38.52%),SVK(45.11%)
	4.18	3.41	3.46	2.27	4.09	2.83	3.91	3.49	
	1.34%	1.34%	68.12%	1.34%	53.15%	14.31%	9.60%	11.05%	

CHI	2.17	1.75	1.81	1.17	3.34	3.19	5.00	2.29	FRA(37.89%),SPA(62.11%)
	3.26	2.25	3.81	2.75	3.85	3.51	5.50	3.53	
	50.55%	28.40%	110.76%	135.47%	15.30%	10.09%	10.09%	54.59%	

BRN	1.79	0.76	2.62	1.60	3.84	2.50	3.86	1.43	FRA(40.11%),SPA(22.70%),ICE(37.20%)
	3.50	1.93	4.26	2.24	4.87	3.17	4.89	3.40	
	96.04%	155.65%	62.75%	40.57%	26.82%	26.82%	26.82%	138.33%	

AUS	1.72	0.87	1.54	1.61	1.33	1.52	2.86	1.14	FRA(6.64%),SPA(83.55%),NOR(0.99%),SVK(8.81%)
	3.22	2.41	2.89	3.01	3.48	2.84	5.93	2.88	
	87.42%	176.56%	87.42%	87.42%	161.13%	87.42%	107.65%	152.38%	

**Table 3:** Cross-efficiency evaluation.

TEAM providing weights										
TEAM	FRA	DEN	SPA	CRO	ICE	HUN	NOR	KOR	SVK	score (ranking)
FRA	1	1	1	1	1	1	1	1	1	1 (1)
DEN	1	1	1	1	1.20	1.09	1.08	1.34	1	1.07 (2)
SPA	1.08	1.21	1	1.03	1.00	1	1.38	1	1	1.07 (2)
SWE	1.32	1.38	1.30	1.28	1.33	1.38	1.52	1.40	1.37	1.37 (17)
CRO	1.09	1.15	1.08	1	1.27	1.15	1.11	1	1.08	1.10 (5)
ICE	1.22	1.27	1.21	1.34	1	1	1.36	1.50	1.18	1.23 (9)
HUN	1.24	1.39	1.23	1.28	1.10	1	1.50	1.28	1.26	1.25 (11)
POL	1.20	1.22	1.20	1.28	1.16	1.14	1.08	1.32	1.12	1.19 (6)
NOR	1.22	1.20	1.22	1.38	1.23	1.13	1	1.50	1.04	1.21 (8)
SRB	1.32	1.32	1.28	1.20	1.71	1.40	1.50	1.29	1.24	1.36 (16)
GER	1.21	1.16	1.19	1.24	1.27	1.44	1.16	1.45	1.17	1.26 (13)
ARG	1.31	1.45	1.27	1.26	1.24	1.22	1.54	1.24	1.30	1.31 (15)
KOR	1.33	1.39	1.29	1.18	1.31	1.10	1.42	1	1.15	1.24 (10)
EGY	1.41	1.51	1.36	1.37	1.53	1.51	1.61	1.62	1.39	1.48 (18)
ALG	1.90	1.83	1.88	2.07	1.74	2.45	1.77	2.58	2.01	2.03 (23)
JPN	1.56	1.65	1.60	1.53	1.32	1.45	1.87	1.77	1.82	1.62 20)
SVK	1.10	1.14	1.10	1.08	1.12	1.05	1	1.07	1	1.07 (2)
AUT	1.26	1.26	1.29	1.41	1.19	1.13	1.16	1.53	1.21	1.27 (14)
ROU	1.22	1.30	1.18	1.26	1.15	1.18	1.39	1.31	1.22	1.25 (11)
TUN	1.59	1.55	1.58	1.68	1.70	1.91	1.45	2.03	1.55	1.67 (21)
BRA	1.19	1.24	1.16	1.14	1.36	1.25	1.14	1.22	1.08	1.20 (7)
CHI	1.48	1.61	1.43	1.51	1.20	1.57	1.74	1.66	1.68	1.54 (19)
BRN	1.70	1.83	1.64	1.70	1.35	1.40	2.21	1.63	1.70	1.68 (22)
AUS	2.29	2.44	2.12	2.15	2.21	2.13	2.63	1.93	1.98	2.21 (24)
